# Insights on the Multifunctional Activities of Magnolol

**DOI:** 10.1155/2019/1847130

**Published:** 2019-05-23

**Authors:** Jianhong Zhang, Zhixi Chen, Xianhua Huang, Weimei Shi, Rui Zhang, Meiling Chen, Hao Huang, Longhuo Wu

**Affiliations:** ^1^Department of General Surgery, First Affiliated Hospital of Gannan Medical University, Ganzhou 341000, China; ^2^College of Pharmacy, Gannan Medical University, Ganzhou 341000, China; ^3^Department of Oncology, Maternity and Child Healthcare Hospital of Jiangxi Province, Nanchang 330006, China

## Abstract

Over years, various biological constituents are isolated from Traditional Chinese Medicine and confirmed to show multifunctional activities. Magnolol, a hydroxylated biphenyl natural compound isolated from* Magnolia officinalis*, has been extensively documented and shows a range of biological activities. Many signaling pathways include, but are not limited to, NF-*κ*B/MAPK, Nrf2/HO-1, and PI3K/Akt pathways, which are implicated in the biological functions mediated by magnolol. Thus, magnolol is considered as a promising therapeutic agent for clinic research. However, the low water solubility, the low bioavailability, and the rapid metabolism of magnolol dramatically limit its clinical application. In this review, we will comprehensively discuss the last five-year progress of the biological activities of magnolol, including anti-inflammatory, antimicroorganism, antioxidative, anticancer, neuroprotective, cardiovascular protection, metabolism regulation, and ion-mediating activity.

## 1. Introduction

**Figure 1:**
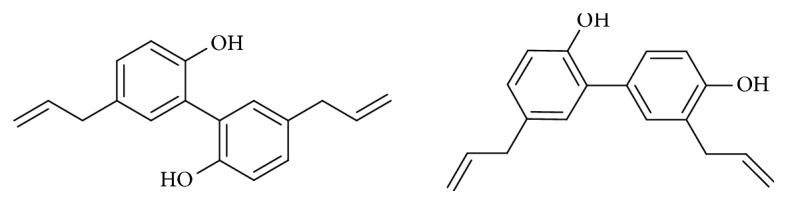
The chemical structures of magnolol (left) and honokiol (right).

Magnolol (5,5'-diallyl-2,2'-dihydroxybiphenyl) (Figure 1) is a polyphenolic binaphthalene compound and a structural isomer of honokiol. Both magnolol and honokiol are isolated from the stem bark of a traditional Chinese herbal medicine* Magnolia officinalis*, which has been used for management of nervous disturbance, abdominal distention or disorders, gastrointestinal food stagnancy, and coughing and dyspnea [1]. Magnolol has showed a wide spectrum of beneficial activities, including anti-inflammation [2, 3], antimicroorganism [4, 5], antioxidation [6, 7], antiangiogenesis [8, 9], anticancer [10–12], neuroprotection [13, 14], cardiovascular protection [15, 16], and lipolysis activities [17, 18]. However, there are still some differences between magnolol and honokiol in safety and toxicology, which have been reviewed by Sarrica et al. (2018)[19]. In this review article, the biological activities of magnolol will be discussed comprehensively.

## 2. Metabolism and Pharmacokinetic Features of Magnolol

Cytochrome P450 monooxygenase (CYP) is the typical drug-metabolizing enzyme (phase I metabolism). The ability of magnolol to interact with drugs has been determined by investigating its effects on CYP enzymes. Magnolol is reported to inhibit the activity of CYP1A with an IC_50_ value of 1.62 *μ*M, of CYP2C with an IC_50_ value of 5.56 *μ*M, and of CYP3A with an IC_50_ value of 35.0 *μ*M. Dixon plot research in rat liver microsomes (RLM) shows that magnolol inhibits CYP1A with* K*_*i*_ value of 1.09-12.0 *μ*M in uncompetitive-dependent manner and CYP2C and CYP3A with* K*_*i*_ values of 10.0-15.2 *μ*M and 93.7-183 *μ*M, respectively, in competitive-dependent manner [20]. In contrast, magnolol inhibits CYP2C enzyme-catalyzed hydroxylation with IC_50_ value of 41.48 *μ*M, CYP2D6 with IC_50_ value of 65.42 *μ*M, CYP2E1 with IC_50_ value of 67.93 *μ*M, CYP3A4 with IC_50_ value of 52.36 *μ*M, and CYP2B6 with IC_50_ value of 28.68 *μ*M [21]. This suggests that magnolol might potentially interact with coadministrated drugs metabolized by CYP enzymes pharmacologically. The direct effects of magnolol on hepatic CYP1A and CYP2C-mediated metabolism have been investigated and found that magnolol inhibits CYP1A (phenacetin as the substrate, IC_50_ value of 19.0 *μ*M) more potently than CYP2C (diclofenac as the substrate, IC_50_ value of 47.3 *μ*M) [22] (Table 2).

The value of mean peak plasma concentration of magnolol reaches 426.4 ± 273.8 ng/mL after oral administration, and the bioavailability of magnolol reaches 17.5 ± 9.7% [23]. However, the absolute bioavailability of magnolol is only 4%. Magnolol is predominantly distributed in liver, kidney, brain, lung, and heart. And the liver contains the highest contents of magnolol and magnolol-glucuronides [24]. In addition, two main metabolites, isomagnolol and tetrahydromagnolol, have been identified and predicted to exhibit a potential synergistic effect with magnolol or other molecules acting on cannabinoid (CB) receptors [25]. The metabolites of magnolol increase dramatically after repeated doses, indicating that they are implicated in the induction of metabolic enzymes [26].

Magnolol is often available for human in daily life because it is the major component of extract, which adds to mints and gums. It has been estimated that the safe dose of magnolol available for teenage is up to 1.64 mg/kg per day. The extensive research indicates that magnolol undergoes glucuronidation (phase II metabolism) before its elimination in human and rats, as indicated by transferring glucuronic acid from UDP-glucuronic acid to magnolol and making it more soluble and excreted by the kidney [27]. Such glucuronidation involves several UDP-glucuronosyltransferase (UGTs) isoforms, including UGT1A1, UGT1A3, UGT1A7, UGT1A8, UGT1A9, UGT1A10, and UGT2B7. Of which UGT2B7 is the major enzyme responsible for magnolol glucuronidation in human liver microsomes (HLM). In addition, UGT1A10 and UGT2B7 are the two major contributors in human intestine microsomes (HIM) [1]. Glucuronides of *β*-estradiol or SN-38 generated in microsomal or HeLa1A1 cell incubations have been quantified, and magnolol shows inhibitory effects (*β*-estradiol: IC_50_ value of 36.8 *μ*M and 22.6 *μ*M, respectively; SN-38: IC_50_ value of 13.2 *μ*M and 16.4 *μ*M, respectively) [28] (Table 2). In addition, UGT1A7 and UGT1A9 have been selectively inhibited on 4-methylumbelliferone (4-Mu) by magnolol with Ki values of 0.487 *μ*M and 0.048 *μ*M, respectively [29]. Thus, magnolol is considered as an atypical substrate of UGT. This indicates that magnolol can show competitive activity with other drugs for interacting with UGT. It has been demonstrated that magnolol in human can inhibit the glucuronidation of propofol, which has considered the biomarker of UGT1A9 reaction. Thus, magnolol has been proposed to prolong the anesthesia time. However, it has been found that magnolol-mediated inhibition of propofol glucuronidation varies among species dramatically, including Bama pigs, cynomolgus macaques, mice, and rats [30].

Due to the low water solubility and quick metabolism, magnolol has a low bioavailability and limits its applications in clinical development. The mixed micelles containing pluronic F127 and L61 for delivering magnolol might be a well strategy for resolving its problem of poor solubility and bioavailability [31]. Similarly, solid dispersion of magnolol with polyvinylpyrrolidone K-30 (PVP) has been shown to increase water solubility of magnolol from 12 to 105 *μ*g/mL, when PVP concentration increases from 0 to 100 *μ*M. The pharmacokinetic parameters indicate that the AUC_0-*t*_ and *C*_max_ of magnolol in solid dispersion with PVP are 80.1% and 44.7%. Additionally, the AUC_0-*t*_ and *C*_max_ of magnolol sulfates or glucuronides are 142.8% and 126.4%, respectively, compared to magnolol pure compound [32]. Furthermore, magnolol-loaded microparticles with size value of 3.73 ± 0.41 *μ*M prepared by single emulsion method from a polyketal polymer have exhibited good biocompatibility and approached macrophage-mediated phagocytosis and pulmonary drug delivery [33].

## 3. Anti-Inflammatory Activity

**Figure 2:**
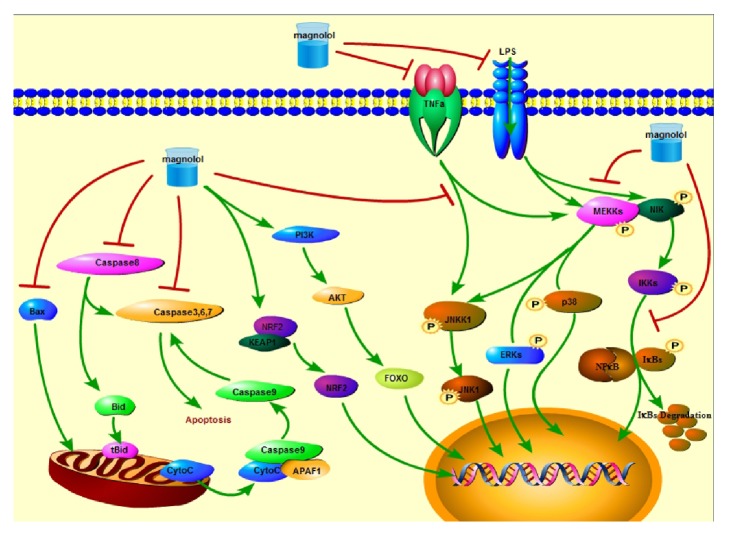
Magnolol inhibits NF-*κ*B and MAPK signaling pathways and apoptosis and activates NRF2/KEAP1 and PI3K/Akt/FOXO signaling pathways. Stimulators, such as LPS and TNF*α*, induce activation of NF-*κ*B and MAPK signaling pathways. Magnolol shows inhibitory effects on NF-*κ*B and MAPK signaling pathways, inhibits mitochondrial apoptosis, but upregulates the activities of NRF2/KEAP1 and PI3K/Akt/FOXO signaling pathways.

Peroxisome proliferator-activated receptor *γ* (PPAR*γ*) has been demonstrated to be capable of ameliorating lipopolysaccharide (LPS)-induced inflammatory activity to suppress the activation of nuclear factor-*κ*B (NF-*κ*B) signaling and the expression of its downstream proinflammatory cytokines in airway epithelial cells and neutrophilia [2]. Magnolol has been investigated for protecting acute lung injury (ALI) and proved to greatly upregulate the expression of PPAR*γ* and suppress the expression of NF-*κ*B signaling, cyclooxygenase-2 (COX-2), and inducible nitric oxide synthase (iNOS) and the production of ROS in bronchoalveolar lavage fluid, resulting in improvement of lung edema and polymorphonuclear neutrophil infiltration [34] (Table 1). Lung inflammation plays a critical role in development of asthma and COPD (chronic obstructive pulmonary disease). Magnolol has been demonstrated to inhibit the increased expression of intercellular adhesion molecule 1 (ICAM-1) induced by tumor necrosis factor *α* (TNF*α*) in A549 cells through suppression of NF-*κ*B/ mitogen-activated protein kinase (MAPK) signaling pathway, as evidenced by downregulation of phosphorylation of NF-*κ*B, p38, extracellular regulated protein kinases 1/2 (ERK1/2), and stress-activated protein kinase (SAPK)/ c-Jun N-terminal kinase (JNK) [35] (Figure 2).

Consistently, magnolol attenuates the expression of TNF*α*, Interleukin 1*β* (IL-1*β*), and IL-12 induced by dextran sulphate sodium (DSS) through downregulation of NF-*κ*B and upregulation of PPAR*γ* expression in ulcerative colitis (UC) mice. In addition, magnolol significantly upregulates the expression of ZO-1 and occludin in DSS-induced UC mice [3]. Tryptophan metabolism greatly contributes to the pathogenesis and therapeutics of inflammatory bowel disease (IBD). Magnolol exhibits protective activity against DSS-induced UC mice with the possible molecular mechanism that involves augment of aryl hydrocarbon receptor (AHR) activation through enhancement of tryptophan metabolites production, which significantly suppresses the colonic inflammation [36]. In 2,4,6-trinitrobenzene sulfonic acid (TNBS)-induced rats colitis, magnolol reduces the activity of colonic myeloperoxidase, the levels of proinflammatory cytokines, and the mRNA expression of toll-like receptor 4 (TLR4). These might be associated with attenuation of NF-*κ*B signaling [37].

In LPS-induced mice uterine epithelial cells (MUECs), magnolol significantly suppresses the expression of TNF*α*, IL-6, p-ERK, p-JNK, and p-p38. These might be associated with inhibition of TLR4-regulated NF-*κ*B signaling and MAPK signaling [38] (Table 1). Similarly, it has been demonstrated that magnolol significantly ameliorates the activity of LPS-induced IL-1*β*, TNF*α*, and IL-6* in vivo* and* in vitro*. In addition, magnolol also decreases the expression of p-p65, p-I*κ*B*α*, p-ERK, p-p38, and p-JNK in LPS-triggered mouse mammary epithelial cells (MMECs) through suppression of TLR4/NF-*κ*B/MAPK signaling pathway [39] (Figure 2). LPS-induced inflammatory and oxidative stresses have been shown to be related to sepsis-triggered dysmotility. Magnolol can significantly accelerate intestinal transit, improve circular muscle contraction, and ameliorate the morphology changes of interstitial cells of Cajal (ICCs) in male gerbils stimulated by LPS. The possible molecular mechanism might be related to the protective activity of magnolol against sepsis-induced decreased expression of SCF/p-c-kit and superoxide dismutase (SOD) and increased expression of iNOS/NO and malondialdehyde (MDA) [40].

In renal I/R injury, magnolol has been found to inhibit the increased expression of TNF*α*, IL-1*β*, IL-6, Bax, and the proapoptotic MAPK (p38 and JNK) phosphorylation and enhance the decreased expression of IL-10, Bcl-2, and the prosurvival Akt and ERK1/2 phosphorylation, resulting in inhibition of apoptosis extrinsically and intrinsically [41]. CB receptors, belonging to the G-protein coupled receptor family, have two subtypes CB1 and CB2. CB2 activation is linked to analgesic and anti-inflammatory activities. Magnolol acts as a partial agonist for CB2 selectively with EC_50_ value of 3.28 *μ*M [25]. Accordingly, magnolol has been demonstrated to be a ligand for CB2 receptor docking at the binding site with EC_50_ value of 1-3 *μ*M [42].

## 4. Antimicroorganism Activity

The release of LPS from* Porphyromonas gingivalis* has been involved in the inflammation-induced periodontitis development. In LPS-triggered RAW 264.7 macrophages, magnolol abrogates the inflammatory responses, as evidenced by downregulation of NF-*κ*B signaling, activation of NF-E2-related factor 2 (Nrf-2)/heme oxygenase-1 (HO-1) signaling, and suppression of iNOS, COX-2, Prostaglandin E2 (PGE_2_), and NO expression. In addition, administration of p38 MAPK and reactive oxygen species (ROS) activators may reverse the effects of magnolol [4] (Table 1).* Streptococcus mutans* (MT8148), known as a cariogenic bacterium, has been reported to induce a low pH environment and form a biofilm. Magnolol has been demonstrated to exert bactericidal activity and prevent and manage dental caries by penetrating biofilm and sterilizing* S. mutans* dose-dependently. In addition, magnolol shows a very low toxicity for gingival epithelial cells at tested concentrations, compared to chlorhexidine (CHX) [43].

*Propionibacterium acnes* and* Propionibacterium granulosum* are the two major bacteria responsible for acne. Magnolol and honokiol have been demonstrated to inhibit significantly both of them with the minimum inhibitory concentrations (MIC) values of 9 *μ*g/mL and 3-4 *μ*g/mL, respectively, by employing the disk diffusion method and a twofold serial dilution assay. Furthermore, both magnolol and honokiol do not produce any side effects in human skin primary irritation test [5].* Aeromonas hydrophila* contributes to pathogenic infections to human and animals through releasing a pore-forming toxin aerolysin, which has become the potential target for drug discovery. Magnolol has been shown to inhibit the hemolytic activity of supernatants from* A. hydrophila* culture through suppression of aerolysin encoding gene* aerA* transcription [44] (Table 1).

Grass carp (*Ctenopharyngodon idella*) often suffers from hemorrhagic disease induced by grass carp reovirus (GCRV). Magnolol has been shown to suppress GCRV replication in CIK cells probably through induction of interferon regulatory factor (IRF)7 and type I IFN (IFN-I) expression. Magnolol significantly promotes IL-1*β* expression but fails to activate NF-*κ*B signaling pathway in GCRV-infected CIK cells [45]. Furthermore, magnolol can effectively improve the resisting ability of grass carp against GCRV and attenuate GCRV-induced cell apoptosis through decreasing the activity of caspase-3, -8, and -9 [46].

## 5. Antioxidative Activity

The antioxidative activity of magnolol has been confirmed by protection from ischemic injury in neurons in stroke animal models (SAM), as shown by suppressive expression of nitrotyrosine, 4-hydroxy-2-nonenal (4-HNE), iNOS, and p-p38 MAPK signaling and downregulation of inflammatory cytokines, including IL-1*β*, TNF*α*, and IL-6 [6] (Figure 2). In the assays of the inhibited autoxidation of cumene and styrene, magnolol has been shown to trap 4 peroxyl radicals with a* K*_*inh*_ value of 6.1 × 10^4^ /M/s in chlorobenzene and of 6.0 × 10^3^ /M/s in acetonitrile. Comparatively, more than 2 peroxyl radicals are trapped by honokiol. This difference is due to a combination of intramolecular hydrogen bonding in magnolol [47]. The review of magnolol and honokiol on their multifunctional antioxidative activity for dermatologic disorders has been made by Shen (2010) [48].

Increased oxidative stress contributes to the development of obesity-related metabolic disorders. Magnolol has been demonstrated to decrease ROS production, upregulate the expression of UCP1, Cd137, Prdm16, Cidea, Tbx1, PGC-1*α*, CPT1, ACSL1, SIRT1, and PLIN, and downregulate the expression of FAS and SREBP1, resulting in browning of 3T3-L1 adipocytes, enhancing lipolysis and thermogenesis, and repressing oxidative stress through activation of AMP-activated protein kinase (AMPK), PPAR*γ*, and protein kinase A (PKA) signaling pathways [7] (Table 1). Acrolein, a neurotoxin, can induce neurodegenerative disorders through inducing oxidative stress and activating MEK/ERK signaling and mitochondrial caspases, leading to apoptosis in neuroblastoma SH-5Y5Y cells. Magnolol pretreatment effectively attenuates acrolein-mediated oxidative stress through decreasing the production of ROS and inhibiting JNK/mitochondrial caspases, PI3K/MEK/ERK, and PI3K/Akt/FOXO1 signaling pathways [49] (Figure 2). The mechanism of bleomycin-induced pulmonary fibrosis is currently believed to be related to oxidative and inflammatory stresses. Due to antioxidative and anti-inflammatory activity, magnolol has been shown to partly reverse the effects of bleomycin on fibrotic process, as evidenced by downregulation of hydroxyproline content, myeloperoxidase (MPO) activity, and TNF*α* and transforming growth factor *β* (TGF*β*) expression and upregulation of SOD expression [65].

On the other hand, aristolochic acids (AA) are nephrotoxic agents, due to their induction of oxidative stress and DNA adducts formation. Magnolol has been found to ameliorate the AA-induced oxidative stress and cell apoptosis in HK-2 cells. However, magnolol worsens overall cell viability in AA-pretreated cells through induction of necrosis, but not autophagy, paraptosis, or accelerated senescence, by causing cell cycle arrest at G1 phase [66]. There is some uncertainty in the interaction between magnolol and drugs, which influences cell viability.

## 6. Anticancer Activity

The anticancer activity of magnolol has been intensively studied in colon and liver cancer cells [67], lung squamous carcinoma CH27 cells [68], L5178Y-ML25 lymphoma [69], and B16-BL6 melanoma [69]. Pretreatment of combination of magnolol with honokiol significantly decreases cell viability and proliferation and increases cell apoptosis in human epidermoid carcinoma A431 cells [10]. In U87MG and LN229 human glioma cells, magnolol, coadministrated with honokiol, has been demonstrated to synergistically inhibit the expression of cyclin A, cyclin D1, and cyclin-dependent kinase (CDK) 2, CDK4, and CDK6, resulting in cell cycle arrest. These might be associated with decreased expression of p-PI3K, p-Akt, p-p38, p-JNK, and Ki67 and increased expression of p-ERK. In addition, combination of magnolol with honokiol can significantly induce autophagy and apoptosis [11]. In androgen insensitive human prostate cancer DU145 and PC3 cell lines, magnolol significantly inhibits the expression of cyclin A, cyclin B1, cyclin D1, cyclin E, CDK2, CDK4, and pRBp107 and enhances the expression of pRBp130, leading to cell cycle alternations. However, the proteins expression of p16INK4a, p21, and p27 are not changed apparently by magnolol after 24 hours administration [12].

Magnolol at lower doses can cause cell cycle arrest at G_0_/G_1_ phase, decrease the expression of cyclin D1, cyclin A, and CDK2, and increase the expression of p21/Cip1 in human glioblastoma cancer (U373) cells [50] (Table 1). At higher dose (100*μ*M), magnolol increases the expression of p27/Kip1 and induces cell apoptosis in U373 cells, accompanied by increased phosphorylation of cSrc, ERK, p38, and Akt, but not JNK [51]. In human gallbladder carcinoma (GBC) cells, magnolol arrests cell cycle at G_0_/G_1_ phase and triggers mitochondria-dependent apoptosis through upregulation of p21 and p53 expression and downregulation of cyclin D1, CDC25A, and CDK2 expression [70]. In cholangiocarcinoma (CCA) cells, magnolol significantly inhibits cell growth, migration, and invasion, accompanied by decreased expression of Ki67, proliferating cell nuclear antigen (PCNA), matrix metalloproteinases 2 (MMP-2), MMP-7, and MMP-9. In addition, magnolol causes cell cycle arrest at G1 phase and downregulates the expression of Cyclin D1, p-I*κ*B*α*, and p-p65 [52] (Table 1).

In nonsmall cell lung cancer A549, H441, and H520 cell lines (NSCLC), magnolol selectively induces DNA fragmentation, decreases mitochondrial membrane potential (Δ*ψ*m), and inhibits cell proliferation. The underlying mechanism might be linked to the release of Bax, Bid, and cytochrome* c* from mitochondria, but inactivation of caspase-3, -8, and -9. However, there is no obvious cytotoxicity of magnolol to normal human bronchial epithelial cells. These indicate an apoptotic pathway induced by magnolol in not caspase-dependent but endonuclease G and cleaved poly(ADP-ribose) polymerase-dependent manner. In addition, magnolol activates the expression of p38 and JNK and attenuates the activity of PI3K/Akt and ERK1/2 in A549 cells [71]. Magnolol has been shown to inhibit the proliferation of NSCLC cell lines, PGCL3, SK-MES-1, NCI-H460, A549, NCI-H1299. Magnolol causes cell cycles arrest at G2/M phase through inhibition of microtubule polymerization and induces cell apoptosis in a p53-independent manner and cell autophagy in an Akt/mTOR-dependent manner [72].

Invasion and metastasis are the two main factors of cancer cells in response to management failure. Magnolol significantly suppresses the invasive activity through downregulation of NF-*κ*B/MMP-9 signaling pathway in human breast cancer cell lines, including MDA-MB-231 cells [73]. Cancer cachexia is a complex metabolic syndrome that is featured by body weight loss, skeletal muscle atrophy, anorexia, and inflammation. Combination of magnolol with chemotherapeutic drugs has been developed to improve skeletal muscle atrophy and inhibit body weight loss in bladder cancer in mice. These might be associated with inhibiting the formation of myostatin and activin A, increasing the activity of p-Akt and p-FOXO3, enhancing the expression of IGF-1, p-mTOR, p-p70S6K, and p-4EBP-1, and decreasing the activity of the proinflammatory cytokines [53] (Table 1).

Angiogenesis is closely related to cancer cells invasion and metastasis and promotes cancer development. Vascular endothelial growth factor (VEGF), a critical mediator of angiogenesis, promotes cells proliferation, migration, and survival through interacting with its receptors, such as VEGFR-1 and VEGFR2. Magnolol can significantly inhibit angiogenesis through decreasing tube formation of HUVECs induced by hypoxia and VEGF in chicken chorioallantoic membrane and matrigel plug. In human bladder cancer cells (T24), magnolol decreases the production of H_2_O_2_ and the expression of HIF-1*α* and increases the degradation of HIF-1*α*. In addition, magnolol acts as an antagonist to directly bind to VEGFR2, leading to attenuation of Akt/mTOR/p70S6K/4E-BP-1 kinase signaling pathways in hypoxic T24 cells and tumors [74]. In HUVECs, magnolol inhibits VEGF-stimulated proliferation, chemotactic motility, tube formation, and vessel sprouting through blocking the activity of MEK/ERK and PI3K/Akt signaling pathways, but not Src and focal adhesion kinase (FAK) signaling pathways, in a Ras-dependent manner [54].

Retinal neovascularization (RNV) may be the neopathy of vascular occlusions, diabetes, vessel regression, and age-related macular degeneration. VEGF undoubtedly promotes the development of and becomes the therapeutic target for RNV [8]. Due to hypoxia, activated microglia stimulates the expression of inflammatory cytokines, which significantly contribute to RNV development. Magnolol has been shownto preserve the astrocyte morphology, inactivate microglia, decrease the expression of inflammatory cytokines, and attenuate HIF-1*α*/VEGF signaling, leading to reduction of RNV [9]. Consistently, magnolol exhibits antiangiogenic activity through induction of ROS and suppressive expression of platelet endothelial cell adhesion molecule (PECAM), which is an endothelial biomarker in mouse embryonic stem (mES)/embryoid body (EB)-derived endothelial-like cells. This might be associated with inactivation of MAPK (p38, JNK, and ERK) and PI3K/Akt/mTOR signaling pathways by magnolol [75].

## 7. Neuroprotective Activity

Dopaminergic neurons degeneration is one of the main pathological characteristics of Parkinson's disease (PD). Magnolol has been shown the neuroprotective activity to reverse the damage induced by 1-methyl-4-phenyl-1,2,3,6-tetrahydropyridine (MPTP) using ^18^F-FP-(+)-DTBZ animal positron emission tomography (PET) [13]. Cholinergic deficits and neuronal dysfunction contribute to Alzheimer's disease (AD). Magnolol has been demonstrated to improve the learning and memory impairment induced by scopolamine (Scop) in mice, as evidenced by restoring the activity of acetyl cholinesterase (AChE), the total contents of SOD and NOS, and the concentration of MDA. These might be associated with the antioxidative activity of magnolol [55] (Table 1). Amyloidogenic protein is also an important causative factor for development of AD, Parkinson's diseases (PD), and type II diabetes. Human calcitonin (hCT), a typical amyloidogenic peptide, aggregates into oligomeric intermediates, which are the most toxic factors during amyloid aggregation and induce cell apoptosis. Magnolol has been shown to not only inhibit the aggregation and amyloid formation of hCT, but also disassemble the preformed aggregation of hCT, through interacting with hCT directly. In addition, magnolol can significantly ameliorate the cytotoxicity caused by hCT aggregates [14].

Recently, a number of research studies report that magnolol exhibits antidepressant activity in rodent models [76–78].* Magnolia officinalis* and its major component magnolol have been demonstrated to shorten the immobility time in the forced swim test (FST) in mice, indicating their antidepressant activity [76]. In the unpredictable chronic mild stress (UCMS) rats model, the expressions of brain-derived neurotrophic factor (BDNF) and glial fibrillary acidic protein (GFAP) are downregulated, and the sucrose consumption and the locomotor activity are significantly attenuated. Magnolol at the doses of 20 and 40 mg/kg shows antidepressant activity and effectively reverses the effects of UCMS [77, 78]. Consistently, magnolol also shows antidepressant activity in an olfactory bulbectomy (OBX) mice model by improvement of impaired hippocampal neurogenesis and increased phosphorylation of extracellular signaling, Akt, ERK, and CREB [79]. In chronic hormone corticosterone (CORT)-induced depressive rats model, the immobility time of FST and tail suspension test (TST) are decreased, along with decreased expression of BDNF, 5-hydroxytryptamine (5-HT), and norepinephrine (NE). Magnolol significantly ameliorates the depressive-like behaviors, decreases CORT levels, and increases the expression of BDNF, 5-HT, and NE in CORT-induced mice [56].

Increasing evidence reports that neuroinflammation, neuroendocrine, and oxidative stress contribute to the development of depression. In chronic mild stress (CMS) mice models, magnolol can significantly downregulate the expression of IL-1*β*, TNF-*α*, IL-6, and MDA, upregulate the levels of SOD and glutathione peroxidase (GSH-Px), and attenuate the activity of hypothalamic-pituitary-adrenal (HPA) signaling pathway [80]. Trimethyltin (TMT) is a useful agent for creating a neurodegenerative model. It has been demonstrated that TMT can induce oxidative stress and trigger cell necrosis/apoptosis. Magnolol pretreatment can significantly reverse the damage induced by TMT, as indicated by decreased production of ROS, inactivation of NF-*κ*B, JNK, and p38 MAPK signaling, and attenuation of microglial activation* in vitro* and* in vivo* [81].

The effects of magnolol on neural injury and blood-brain barrier (BBB) induced by ischemia-reperfusion (I/R) have been investigated. Magnolol, at the doses of 1.4, 7.0, and 35 *μ*g/kg, can significantly decrease cerebral infarct volume, brain water content, and the exudation of Evans blue in mice with I/R. In the primary microglial cells, magnolol has been shown to inhibit the release of NO and the expression of TNF*α* and p65 translocation induced by LPS. In oxygen and glucose deprivation-reperfusion models of the media of brain microvascular endothelial cells (BMECs) and BBB, magnolol significantly ameliorates the expression of TNF*α*, IL-1*β*, and EphA2 phosphorylation and elevates the production of zonula occludens-1 (ZO-1) and occludin [57] (Table 1). In ischemic stroke rat models, magnolol has been shown to ameliorate brain edema, reduce infarct volume, and increase neurological score. This might be associated with the anti-inflammatory activity of magnolol, as indicated by downregulation of IL-1*β*, TNF*α*, Bax, and Ac-FOXO1 expression and upregulation of Bcl-2 and SIRT1 expression [82]. In traumatic brain injury, magnolol can significantly decrease the levels of glycerol and hydroxyl radical and increase the expression of TGF-*β*1 in hippocampus, leading to reduction of infarct volume and neuronal apoptosis [83].

## 8. Cardiovascular Protection Activity

Recently, clinical evidence shows that patients with prehypertension (high-normal blood pressure) are more likely to progress to hypertension than those who are with normal or optimal blood pressure [84]. Magnolol has been demonstrated to restore insulin-mediated activity of Akt and eNOS and of vasolidation of aorta, upregulate the expression of PPAR*γ*, and downregulate the expression of TRB3 in spontaneous hypertensive rats (SHR) and cultured human umbilical vein endothelial cells (HUVECs) [15] (Table 1). The activity of cell adhesion molecules (CAMs) and the attachment of leukocytes to the endothelium are important for cardiovascular disorders. Magnolol has been shown to inhibit the phosphorylation of JNK/p38, the translocation of HuR, the activation of NF-*κ*B, and the expression of vascular CAM-1 (VCAM-1), leading to reduction of leukocyte adhesion in TNF*α*-treated human aortic endothelial cells (HAECs) [58].

Vascular smooth muscle cells (VSMCs) basically reside in quiescent form, and their enhanced proliferation and migration induced by TNF*α* contribute to atherosclerotic and restenotic lesions formation. Magnolol has been demonstrated to cause cell cycle arrest at G_0_/G_1_, downregulate the expression of cyclin D1, cyclin E, CDK2, and CDK4, and attenuate the activity of p-ERK1/2 and NF-*κ*B signaling pathways [16] (Table 1). Similarly, magnolol inhibits the proliferation of VSMCs induced by platelet-derived growth factor (PDGF)-BB through reduction of intracellular ROS and decreased expression of Ras, MEK, and ERK1/2 [85]. In addition, the migration of VSMCs can be strongly inhibited by magnolol through attenuation of cytoskeletal remodeling signaling pathway without affecting the activity of MMPs, as evidenced by suppressive expression of *β*1-integrin, RhoA, Cdc42, p-FAK, and p-MLC20* in vivo* and* in vitro*, leading to inhibition of neointima formation [86].

## 9. Metabolism-Regulating Activity

Combinational activation of retinoid X receptor *α* (RXR*α*) and PPAR*γ* is believed to increase their effects on glucose and lipid metabolism synergistically. Magnolol acts as a dual agonist for activating both RXR*α* and PPAR*γ*. However, there is a biased agonism of magnolol to promote the transcriptional activity of PPAR-response element (PPRE), which is regulated by the heterodimer of RXR*α*:PPAR*γ*, instead of RXR-response element (RXRE) by RXR*α*:RXR*α* [87]. To further investigate the modulating activity of magnolol in lipid metabolism* in vivo*, a transgenic knock-in mice carrying apolipoprotein A5 (APOA5) c.553G>T variant has been established. Magnolol has been found to decrease the levels of the plasma triglyceride in the transgenic mice and increase the expression of lipoprotein lipase (LPL) in 3T3-L1 preadipocytes. These indicate the facilitation of triglyceride metabolism and reduction of hyperlipidemia [18]. Magnolol enhances the differentiation of adipocytes and the uptake of glucose, leading to amelioration of glucose level in 3T3-L1 cells [59]. Potent glucosidase inhibitors are able to retard glucose absorption and reduce blood glucose levels. Magnolol shows the inhibitory activity on *α*-glucosidase with IC_50_ value of 2.0 *μ*M [62]. Protein tyrosine phosphatase-1B (PTP1B) is a major nontransmembrane phosphotyrosine phosphatase and acts as a negative regulator of the insulin signaling pathway. It has been demonstrated that magnolol inhibits the activity of PTP1B with IC_50_ value of 24.6 *μ*M [63]. Peroxisome proliferator-activated receptor gamma (PPAR*γ*) agonists are used for the treatment of type 2 diabetes and metabolic syndrome. Being an agonist, magnolol activates PPAR*γ* with* Ki* value of 2.04 *μ*M [64] (Table 2). Magnolol has been reported to induce lipolysis in lipid-laden RAW 264.7 macrophages through attenuation of adipose differentiation-related protein (ADRP) expression in a cAMP-PKA-independent manner [17] (Table 1).

Liver X receptors (LXR) participate in the regulatory processes of many physiological activities, including metabolism of glucose, cholesterol, and fat. It has been demonstrated that magnolol can combine with LXR*α* dose-dependently and regulate its downstream genes expression in HepG2 and THP-1 cell lines [88]. High-fat diet (HFD) consumption has been linked to activation of hepatic steatosis and liver dysfunction. LXR*α* activation leads to increased expression of sterol regulatory element binding protein -1c (SREBP-1c) and lipogenic genes. These can be abrogated by treatment of magnolol and honokiol through upregulation of AMPK, which exhibits inhibitory activity to LXR*α*-SREBP-1c signaling pathway [89].

Consistently, activation of Akt/AMPK/PPAR*α* signaling and attenuation of MAPK/NF-*κ*B/SREBP-1c signaling by magnolol have been linked to its effects against steatosis and hyperlipidemia, as indicated by suppression of oleic acid (OA)-induced triglyceride (TG) accumulation, ROS production, and TNF*α* expression in HepG2 cells [90]. In HFD-fed mice, magnolol can significantly decrease the weight of white adipose tissue (WAT) and the size of adipocyte and reverse insulin resistance. These might be the results of augment of energy expenditure and adipose fatty acid oxidation and attenuation of the activity of fatty acid synthase and the expression of genes relating to adipocyte differentiation, fatty acid synthesis, and desaturation in WAT. In addition, magnolol has been shown to ameliorate the expression of proinflammatory genes and enhance the level of the plasma IL-10 [91].

Methylglyoxal (MG) is a precursor of advanced glycation end-products (AGEs), which contribute to insulin resistance. In RIN-m5F *β*-cells, MG causes decreased viability and impaired insulin secretion, which can be reversed by magnolol. In addition, magnolol is involved in promotion of *β*-cell survival and functions, as indicated by increased expression of Ins2, PDX1, p-AMPK, SIRT1, and PGC-1*α*. Furthermore, magnolol enhances the activity of glyoxalase I and attenuates the expression of MG-mediated protein adducts, leading to protection against MG-induced glycation in RIN-m5F *β*-cells [92].

## 10. Ion-Mediating Activity

Magnolol has been reported to show different responses to Ca^2+^ signaling in various cell types. Magnolol can increase cytoplasmic free Ca^2+^ through induction of Ca^2+^ mobilization in human neuroblastoma SH-SY5Y cells and primary rat cortical neurons [93] and enhance the cytosolic Ca^2+^ concentration through stimulatory release of Ca^2+^ from internal stores and external influx across the plasma membrane [94]. Magnolol also induces the rise of cytosolic Ca^2+^ concentration through stimulation of phospholipase C (PLC)-dependent release of Ca^2+^ partially from endoplasmic reticulum and induction of Ca^2+^ entry by PKC-mediated store-managed Ca^2+^ mobilization, leading to Ca^2+^ signaling-dependent cell death in oral cancer cells [95]. On the other hand, magnolol blocks the influx of Ca^2+^ through voltage-gate Ca^2+^ channels (VGCC), leading to relaxation of porcine tracheal smooth muscle [96]. The inhibitory effects of magnolol on uterine contraction might be abolished by abrogation of external Ca^2+^ influx induced by PGF(2*α*) and high K^+^[97]. And, L-type Ca^2+^ channel activity is attenuated by magnolol, which inhibits colonic smooth muscle contraction [98]. This alternation of intracellular Ca^2+^ mobilization induced by magnolol through VGCC is also confirmed in guinea pig [99].

In enterotoxigenic* Escherichia Coli* (ETEC)-triggered diarrhea mice, magnolol has been found to increase the levels of Cl^−^ and K^+^ and the mRNA expression of calmodulin 1 (CaM), potassium large conductance calcium-activated channels (BK)*α*, and BK*β*3 and decrease the mRNA expression of IP3R1, PKC, potassium small conductance calcium-activated channels (SK)1, SK2, SK3, SK4, and BK*β*4. However, magnolol does not influence the mRNA expression of CaMKII*α*, CaMKII*β*, IP3R2, IP3R3, BK*β*1, BK*β*2, and ryanodine receptor (RYR) 1 [100].

## 11. Clinical Prospective

The emerging multidrug-resistant bacteria cause life-threatening infections.* Staphylococcus aureus* shows resistance to wide spectrum *β*-lactam antibiotics, such as methicillin and oxacillin. Infection caused by methicillin-resistant* S. aureus* (MRSA) poses a serious global issue. Magnolol and honokiol have been reported to show antibacterial activity against MRSA, methicillin-susceptible* S. aureus* (MSSA), and* S. aureus* dose-dependently. Importantly, magnolol exhibits synergistic activity with oxacillin against 13 clinical isolated MRSA. The underlying mechanism might be associated with downregulation of the resistant genes expression in* S. aureus*, including* mecA*,* mecI*,* femA*, and* fem B* [101]. In addition, magnolol significantly decreases ROS production and inflammatory cytokines expression in response to* S. aureus* in mouse macrophages. The internalization of* S. aureus* by human alveolar epithelial cells is also attenuated by magnolol [102]. Magnolol does not show any antagonistic effects on the 10 MRSA strains, when it is combined with the antibacterial agents synergistically. MIC_50_ of magnolol in combination decreases from 16 mg/L to 1-4 mg/L and the antibacterial agent decreases from 8-128 mg/L to 2-64 mg/L. Importantly, magnolol shows reversal effects on MRSA to resensitize to amikacin and gentamicin [103]. These suggest that magnolol demonstrates the potential for managing *β*-lactam treatments against the resistant strains of infectious bacteria.

*Candida* spp. has been considered as a critical pathogen for nosocomial bloodstream infection. Due to prolonged exposure to azoles,* Candida* spp. with azoles resistance causes therapeutic failure with a high rate of mortality. Magnolol has been shown to prolong the survival of* Candida albicans*-infected nematodes through inhibiting Ras1-cAMP-Efg1 signaling-mediated adhesion, transition from yeast to hypha, and biofilm formation of* C. albicans* [104]. The minimum inhibitory concentration (MIC) of magnolol against* C. albicans* and nonalbicans Candida is 10-40 *μ*g/mL. The BMIC_90_ (minimum inhibitory concentration that inhibits 90% biofilm formation) value of magnolol is 20-160 *μ*g/mL [105]. Magnolol has been found to exhibit synergistic effects with fluconazole to inhibit the activity of* Candida albicans* through induction of higher concentration of intracellular antifungals. The molecular mechanism might be associated with competition of magnolol with ABC transporter Cdr1p substrates and enhancement of the ergosterol biosynthesis pathway [106]. Magnolol can effectively inhibit growth and biofilm formation in oral Candida isolates with MIC value of 16-64 g/mL. The possible mechanism might involve the disruption of cell membrane by magnolol by using confocal scanning laser microscopy and transmission electron microscopy. In addition, molecular docking reveals that magnolol might interact with ergosterol directly in the fungal cell membrane [107]. In a six-month clinical study, unsupervised brushing with a dentifrice containing 0.3% Magnolia extract significantly reduces dental plaque and gingivitis, as indicated by reduction of gingival index (GI) and Quigley and Hein index (QHI) compared to placebo [108]. Consistently, Magnolia chewing gum can also reduce plaque acidogenicity, salivary* mutans streptococci* concentration, and gingival bleeding after 30 days of treatment [109], indicating beneficial effects of magnolol on oral health.

Ligature-induced periodontitis leads to the alveolar bone loss posing a serious oral-health issue in clinical practice. Magnolol can significantly protect against alveolar bone resorption, osteoclast growth on bony surface, and the protein expression of receptor activator of nuclear factor-*κ*B ligand (RANKL). In addition, magnolol also reduces the expression of iNOS, COX-2, MMP-1, MMP-13, ROS, and NF-*κ*B activation in gingival tissues.* In vitro*, magnolol inhibits the growth of* Porphyromonas gingivalis* and* Aggregatibacter actinomycetemcomitans* with both IC_50_ values of about 100 *μ*M, the differentiation of osteoclast from RAW264.7 macrophages induced by RANKL, and the activity of tartrate-resistant acid phosphatase (TRAP) in differentiated osteoclast [61]. The underlying mechanism of magnolol in mediating RNAKL-triggered osteoclast differentiation and bone resorption is related to suppression of NFATc1 nuclear translocation, as evidenced by inhibition of ROS generation, MAKP/c-fos/AP-1 signaling, and NF-*κ*B signaling and increased expression of HO-1 [110].

Magnolol lacks direct antibacterial activity, but it inhibits the enzyme activity of New Delhi metallo-*β*-lactamase-1 (NDM-1) by directly interacting with the catalytic pocket with an IC_50_ value of 7.47 *μ*g/mL and restores the activity of meropenem against* Escherichia Coli* ZC-YN3 with decreased MICs by 4-fold [60]. Magnolol has been reported to inhibit endothelial cell activity during pressure-regulated venous remodeling processes, which play a crucial role in varicose and spider veins development. The possible underlying mechanism might be associated with the antiproliferative and antioxidative activity of magnolol through enhancement of HO-1 signaling and downregulation of cell proliferation, ERK1/2 activity, gelatinase activity, and ROS baseline production. However, the formation of endothelial capillary sprout is not influenced by magnolol [111]. In concanavalin A (ConA)-stimulated transformation from CD4+ T cells to CD4+T helper (Th17) cells in liver, magnolol significantly inhibits this cellular polarization, Th17 cell differentiation, and IL-17A production. In addition, magnolol suppresses the activation of hepatic stellate cells in fibrotic liver, as shown by decreased expression of *α*-smooth muscle actin (*α*-SMA), desmin, TGF*β*1, and activin A. These might be related to partial blockade of Smad3 signaling pathway by magnolol in LX2 cells [112].

Heat stress (HS) adversely affects livestock in summer. It has been reported that HS causes intestinal epithelial cell line (IEC-6) injury and cell cycle arrest at G1 phase, as indicated by increased expression of p21 and p27 and decreased expression of E2F1, pRb, cyclin D1, and CDK4. Magnolol pretreatment protects against HS-triggered injury in IEC-6 cells effectively [113]. Magnolol has been demonstrated to show antiphotoaging activity in UV-irradiated hairless mice, as evidenced by reduction of the average length and depth of wrinkles, downregulation of MMP-1, MMP-9, and MMP-13 expression, and attenuation of MAPKs phosphorylation, including p38, ERK1/2, and JNK [114]. In China, the effects of* Magnolia officinalis* on gastrointestinal motility disorder have been intensively investigated [115].* Magnolia officinalis* improves gastrointestinal motility disorder in rats by increasing the concentration of L-glutamate, L-tryptophan, and serotonin and protecting gastrointestinal barrier. Similarly, magnolol mainly affects 10 major metabolic pathways, of which tryptophan metabolism is the most important pathway associated with gastrointestinal tract. The underlying mechanism might be related to reduction of NO production, attenuation of NO functions in relaxing the gastrointestinal smooth muscle, and promotion of gastrointestinal peristalsis and motility, secretion, absorption of nutrients by increasing serotonin content [116]. Consistently, magnolol enhances the anti-inflammatory capacities in intestine, elongates the villus height and crypt depth, and reduces goblet cell numbers, leading to protection of intestinal mucosal integrity and amelioration of gastrointestinal dysfunction [117].

Magnolol, a natural compound with versatile biological functions, has been demonstrated to be safe with absence of any mutagenic and genotoxic potentials [19, 118]. In addition, magnolol exhibits antimutagenic activity effectively [119]. Magnolia bark extract (MBE), consisting of 94% magnolol, has the oral LD50 value with more than 50 g/kg body weight [118]. No obvious sign of toxicity has been obtained for MBE (60-480 mg/kg b.w./d for 21 days and 60-240 mg/kg b.w./d for 90 days) [120]. However, magnolol (no less than 0.5 mg/kg b.w.) has been reported to prolong the mice tail bleeding time at low dose of 10 mg/kg b.w., indicating the potential for developing an antihemostatic agent. Additionally, magnolol has showed spasmolytic effects on uterine muscle contraction. This makes it possible for magnolol to be used for treatment of gynecological disturbances and dysfunctions associated with an increase in uterine muscular activity. However, the effects of magnolol in pregnancy are still not justified. On perception of safety for natural product, magnolol could be used as a sedative, due to its GABAergic and cannabimimetic activity [121]. Management of abuse/misuse of magnolol should be deeply discussed.

## 12. Concluding Marks

**Figure 3:**
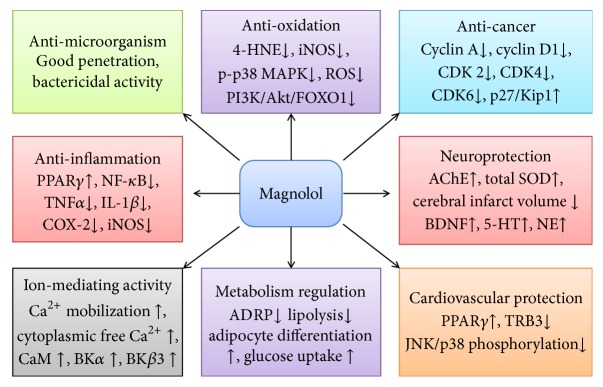
The multifunctional activities of magnolol. Magnolol has been shown to result in anti-inflammation, antimicroorganism, antioxidation, anticancer, neuroprotection, cardiovascular protection, metabolism regulation, and ion-mediating activity.

In this review, we mainly discuss the biological activities of magnolol (Figure 3) in the last 10 years. Mechanistically, magnolol shows inhibitory activity on NF-*κ*B and MAPK signaling pathways and their target genes expression, leading amelioration of inflammation and oxidation. These are available for the strategies in protecting neural and cardiovascular diseases. In addition, magnolol mediates the expression of proliferation-related proteins, which might be the potential targets for managing cancers. Metabolism regulation and ion mediation are the two critical cellular physiological processes, during which imbalanced homeostasis may result in severe pathology and be restored by magnolol. These findings have greatly increased the interest in bridging magnolol to the clinic as a promising therapeutic agent.

However, information in this review on structural modifications of magnolol has been neglected. Unfortunately, the low water solubility, the low bioavailability, and the rapid metabolism of magnolol dramatically limit its clinic application, although many useful approaches have been under investigation. Most studies on the biological activities of magnolol* in vitro* are focusing on the parent form. However, the increasing evidence shows that the parent form of magnolol is not present in the systemic circulation. Therefore, whether the knowledge of magnolol-benefit obtained in the* in vitro* studies can predict the actual effects of magnolol* in vivo* is still unclear. And not even one clinical data has been obtained to show the efficacy of magnolol. To fully rationalize the potential of magnolol, more efforts and clinical trials are needed. In addition, the metabolites of magnolol also exhibit great biological potentials. Investigation on the functions of the metabolites of and the derivatives of magnolol* in vivo* and* in vitro* may lead to exploration of new drugs.

## Figures and Tables

**Table 1:** The multifunctional activities of magnolol.

Category	Models	Doses	Biological activities	Ref.
Anti-inflammation	UC mice	25, 50, and 100 mg/kg	TNF*α*↓, IL-1*β*↓, IL-12↓, PPAR*γ*↑, NF-*κ*B↓, ZO-1↑, occludin↑	[3]
	LPS-induced ALI rats	10 and 20 mg/kg	PPAR*γ*↑, NF-*κ*B↓, COX-2↓, iNOS↓, ROS↓, lung edema↓	[34]
	A549	20 and 50 *μ*mol/L	ICAM-1↓, NF-*κ*B↓, p38↓, ERK1/2↓, SAPK/JNK↓	[35]
	LPS-induced MUECs	12.5, 25, 50*μ*g/mL	TNF*α*↓, IL-6↓, p-ERK↓, p-JNK↓, p-p38↓, NF-*κ*B↓, MAPK↓	[38]
	LPS-induced MMECs	12.5, 25, 50*μ*g/mL	IL-1, TNF*α*↓, IL-6↓, p-p65↓, p-I*κ*B*α*↓, p-ERK↓, p-p38↓, p-JNK↓	[39]

Anti- microorganism	LPS-induced RAW 264.7	5, 10, and 20*μ*M	Nrf-2/HO-1↑, NF-*κ*B↓, iNOS↓, COX-2↓, PGE_2_↓, NO↓	[4]
	MT8148	10, 20, and 50*μ*g/mL	Good penetration and bactericidal activity on MT8148 biofilm	[43]
	Channel catfish	2, 4, 8, and 16*μ*g/mL	Inhibit hemolytic activity of supernatants from *A. hydrophila *culture	[44]

Anti-oxidation	SAM	10 and 30 mg/kg	4-HNE↓, iNOS↓, p-p38 MAPK↓, IL-1*β*, TNF*α*↓, IL-6↓	[6]
	3T3-L1	5, 10, and 20*μ*M	ROS↓, FAS↓,SREBP1↓, UCP1↑, Cd137↑, Prdm16↑, Cidea↑, Tbx1↑, PGC-1*α*↑, CPT1↑, ACSL1↑, SIRT1↑, PLIN↑	[7]
	SH-5Y5Y	8, 16, and 32*μ*M	ROS↓, JNK/mitochondrial caspases↓, PI3K/MEK/ERK↓, PI3K/Akt/FOXO1↓	[49]

Anti-cancer	U87MG and LN229	20 and 40*μ*M	Cyclin A↓, cyclin D1↓, CDK 2↓, CDK4↓, CDK6↓, p-PI3K↓, p-Akt↓, p-p38↓, p-JNK↓, Ki67↓, p-ERK↑	[11]
	U373	10, 20, and 40*μ*M	Arrest at G_0_/G_1_ phase, cyclin D1↓, cyclin A↓, CDK2↓, p21/Cip1↑	[50]
	U373	100*μ*M	p27/Kip1↑, ↑phosphorylation of cSrc, ERK, p38, and Akt.	[51]
	CCA	20 and 40*μ*M	Ki67↓, PCNA↓, MMP-2↓, MMP-7↓, MMP-9↓, CyclinD1↓, p-I*κ*B*α*↓, p-p65↓	[52]
	Mice bladder cancer	10 mg/kg/d	Myostatin↓, activin A↓, p-Akt↑, p-FOXO3↑, IGF-1↑, p-mTOR↑, p-p70S6K↑, p-4EBP-1↑	[53]
	HUVECs	0.1, 0.5, and 1*μ*M	Inhibit VEGF-stimulated proliferation, chemotactic motility, tube formation, and vessel sprouting, MEK/ERK↓, PI3K/Akt↓	[54]

Neuroprotection	Scop-induced mice	15, 25, and 35 mg/kg	AChE↑, total SOD↑, NOS↑, MDA↑	[55]
	CORT-induced rats	50 and 100 mg/kg	FST↑, TST↑, BDNF↑, 5-HT↑, NE↑	[56]
	I/R mice	1.4, 7.0, and 35 *μ*g/kg	Decrease cerebral infarct volume, brain water content, and the exudation of Evans blue	[57]

Cardiovascular protection	SHR and HUVECs	100 mg/kg/d and 10*μ*M	Restore insulin-mediated Akt, eNOS, and aorta vasolidation, PPAR*γ*↑, TRB3↓	[15]
	VSMCs	5, 10, 20, and 30*μ*M	Arrest at G_0_/G_1_, cyclinD1↓, cyclinE↓, CDK2↓, CDK4↓, p-ERK1/2↓, NF-*κ*B↓	[16]
	HAECs	5*μ*M	JNK/p38 phosphorylation↓, HuR translocation↓, NF-*κ*B↓, VCAM-1↓	[58]

Metabolism regulation	RAW 264.7	20, 40, and 60*μ*M	ADRP↓, lipolysis↓	[17]
	3T3-L1	1 and 10*μ*M	Enhances adipocytes differentiation and glucose uptake	[59]

**Table 2:** The inhibitory effects of magnolol on different targets.

Substrate	Enzyme (target) source	IC_50_ (*μ*M)	*Ki* (*μ*M)	EC_50_ (*μ*M)	Ref.
NF	CYP1A	1.62	1.09-12.0	-	[20]
SPZ	CYP2C	0.56	10.0-15.2	-	
KCZ	CYP3A	35	93.7-183	-	

meropenem	NDM-1	6.47 (*μ*g/mL)	-	-	[60]

*-*	*Porphyromonas gingivalis*, *Aggregatibacter actinomycetemcomitans*	100	-	-	[61]

CP55,940	CB2	-	1.44	3.28	[25]

pNP-*α*-G	*α*-Glucosidase	2.0	-	-	[62]

4-hydroxy tolbutamide	CYP2C	41.48	-	-	[21]
dextrorphan	CYP2D6	65.42			
6-hydroxy chlorzoxazone	CYP2E1	67.93			
6-hydroxy testosterone	CYP3A4	52.36			
hydroxybupropion	CYP2B6	28.69			

p-NPP	PTP1B	24.6	-	-	[63]

-	PPAR*γ* (agonist)	-	2.04	1.6	[64]

*β*-Estradiol	UGT1A1	36.8	-	-	[28]
*β*-Estradiol	Hela1A1	22.6	-	-	
SN-38	UGT1A1	13.2	-	-	
SN-38	Hela1A1	16.4	-	-	

4-Mu	UGT1A7	-	0.487	-	[29]
4-Mu	UGT1A9	-	0.048	-	
